# Lack of association between genetic polymorphisms in *IGF1* and *IGFBP3* with twin births in a Brazilian population (Cândido Godói, Rio Grande do Sul)

**DOI:** 10.1590/1678-4685-GMB-2017-0263

**Published:** 2018-11-29

**Authors:** Mariana de Oliveira-Klein, Augusto César Cardoso-dos-Santos, Alice Tagliani-Ribeiro, Nelson Rosa Fagundes, Ursula Matte, Lavinia Schuler-Faccini

**Affiliations:** ^1^ Departmento de Genética, Instituto de Bociências, Universidade Federal do Rio Grande do Sul, Porto Alegre, RS, Brazil; ^2^ Instituto Nacional de Ciência e Tecnologia de Genética Médica Populacional(INaGeMP), Departmento de Genética, Instituto de Bociências, Universidade Federal do Rio Grande do Sul, Porto Alegre, RS, Brazil; ^3^ Serviço de Genética Médica, Hospital de Clínicas de Porto Alegre, Porto Alegre, RS, Brazil

**Keywords:** Insulin-like growth factor, founder effect, microsatellite, reproduction, twinning

## Abstract

Insulin-like growth factor (IGF-1) is an important peptide hormone involved in the reproduction and fetal development of mammals, and it is suggested that it may influence the human twinning rate. This study aimed to test such possible association, investigating the genetic polymorphisms *IGF1* (CA)n and *IGFBP3* rs2854744 in the population from Candido Godoi (CG), a small city located in the South of Brazil that has a high prevalence of twin births. A case-control study was performed comprising a total of 39 cases (representing about 40% of the mothers of twins who were born in CG after 1995) and 214 controls (mothers of non-twin children), 97 of whom were living in CG while 117 were living in Porto Alegre. DNA was extracted from blood leucocytes and genotyping was performed. According to the statistical analyses, there was no significant difference in the frequencies of both studied genetic polymorphisms when comparing case group with control group. Thus, our results pointed to a lack of association between *IGF1* (CA)n and *IGFBP3* rs2854744 polymorphisms and twin births in CG, but further investigations in other populations with different characteristics must be performed to confirm the role of IGF-I in human twinning.

## Introduction

Although genetic factors related to twin births in the human species have been investigated by different approaches (Montgomery *et al.*, 2001; Painter *et al.*, 2010; Tagliani-Ribeiro *et al.*, 2012; Mbarek *et al.*, 2016), there is still a confusing scenario of which genes or alleles may be related to etiology of twinning, because it is a complex phenomenon and different causes may be involved in different situations (Lambalk *et al.*, 1998; Huang *et al.*, 2015; Mardini *et al.*, 2017).

A small city in the South of Brazil, Cândido Godói (CG, latitude 27°45’07”, longitude 54°45’07”), has attracted the attention of researchers and curious observers due to its high rates of natural monozygotic (MZ) and dizygotic (DZ) twin births and because this trait runs among local families (Matte *et al.*, 1996; Tagliani-Ribeiro *et al.*, 2011). Studies previously performed in this population have suggested that the founder effect hypothesis linked to geographical isolation may explain such peculiarity, associated to the finding of a single nucleotide polymorphism (SNP) of *TP53* as a strong risk factor for twinning, possibly due to its important role in blastocyst implantation and *intra utero* embryo survival (Tagliani-Ribeiro *et al.*, 2012). However, it is expected that other factors, including other genetic polymorphisms, contribute with the high twinning rates found in CG.

Besides the p53 pathway, factors related to individual endocrine profiles stand out as having a possible influence in twin births (Montgomery *et al.*, 2001; Rickard *et al.*, 2012). Insulin-like growth factor (IGF-1), for example, has previously been proposed to be associated with twin births in humans (Steinman, 2009), and biochemical and genetic studies have already related such factor to twinning in cattle (Echternkamp *et al.*, 1990, 2004; Kim *et al.*, 2008).

IGF-1 is an important peptide hormone for cellular differentiation and growth, and it is involved in mechanisms related to reproduction and fetal development of mammals(Adam *et al.*, 2000; Echternkamp *et al.*, 2004; Thomas *et al.*, 2016). In human reproduction, IGF-1 acts as a pituitary regulator of follicular growth and potentiates the action of both gonadotrophins, luteinizing hormone, and follicle stimulating hormone (Ohlsson *et al.*, 2009). In the ovary, it regulates the differentiation of granulosa cells and the development of the follicle, and may influence the twinning rate (Lambalk *et al.*, 1998; Erickson and Shimasaki, 2001). Furthermore, traits associated with IGF-1, such as ethnicity and body mass index, vary with twinning ratio (Steinman, 2009; Rickard *et al.*, 2012).

Genetic factors are known to influence IGF-1 levels and individual variation (Rosen *et al.*, 1998; Kwasniewski *et al.*, 2016). The *IGF1* gene has a polymorphic microsatellite of cytosine and adenine nucleotides, (CA)n, which ranges in sizes from 10 to 24 CA repeats, located at the *IGF1* promoter region, approximately 1 kb upstream from the transcription start site (GenBank accession number AB133839.1) (Jernström *et al.*, 2001). In the Caucasian population, the most common allele contains 19 CA repeats (NG_011713.1:g.4248CA[19]), commonly known as (CA)19, which corresponds to fragments of 192bp after amplification (Costalonga *et al.*, 2012). Interestingly, an exception can be found in the results by Kato *et al.* (2003), in which a Caucasian population presented the allele with 18 CA repeats as the most frequent one, and the authors listed the small sample size and population admixture as possible explanations for this discrepancy. The length of CA repeats has been associated with serum IGF-1 levels and to different human phenotypes, as well as to breast and endometrial cancers (Wagner *et al.*, 2005; Kwasniewski *et al.*, 2016), bone disorders (Kim *et al.*, 2002), and others (Costalonga *et al.*, 2012; Kaczmarek *et al.*, 2015).

In turn, the *IGFBP3* gene (which synthesizes insulin-like growth factor binding protein 3) is responsible for transporting about 90% of the circulating IGF-1 and is capable of deregulating the levels of IGF-1 present in the plasma (Al-Zahrani *et al.*, 2005; Ohlsson *et al.*, 2009). There is evidence that the polymorphism rs2854744: C > A (NG_011508.1:g.4797C > A) in *IGFBP3* is a genetic factor influencing the levels of IGFBP-3 and IGF-1, and it has been shown that women homozygous for the A allele have higher levels of circulating IGFBP-3, and consequently, higher levels of circulating IGF-1 (Ali *et al.*, 2003; Al-Zahrani *et al.*, 2005; Costalonga *et al.*, 2009; Ohlsson *et al.*, 2009).

Taking into consideration the role of *IGF1* (CA)n and *IGFBP3* rs2854744 polymorphisms in the metabolism of IGF-1 and in the reproductive context, the aim of this study was to investigate whether such genetic polymorphisms can help to explain the high rates of twin births found in the city of Cândido Godói, in the state of Rio Grande do Sul, Brazil.

## Subjects and Methods

We designed a population-based case-control study with mothers of twins in case group and mothers of singletons in two control groups. The study was approved by the ethics committee of the Hospital de Clínicas of Porto Alegre (HCPA) under protocol #09-359. All procedures contributing to this work complied with the ethical standards of the relevant national and institutional committees on human experimentation and with the Helsinki Declaration of 1975, as revised in 2008.

The case group was formed by 39 women who were mothers of twins and resided in the municipality of Cândido Godói (CG). This number represents about 40% of the mothers of twins born in CG after 1995 (Tagliani-Ribeiro *et al.*, 2011). To compare with the case group, we studied a control group composed by 97 mothers of non-twin children born in the municipality of CG. The characteristics of both case and control groups from CG were already described in Tagliani-Ribeiro *et al.* (2012).

In addition, considering the common ancestral origin of the CG population, we included an external control group to determine the frequency of the investigated alleles in the state of Rio Grande do Sul consisting of 117 mothers of non-twin children living in Porto Alegre (PA), the state capital. The control group from PA had a mean age of 22.6 years and a mean number of pregnancies of 2.5, similar to the control group from CG (Tagliani-Ribeiro *et al.*, 2012).

Taking into account that CG has an increased prevalence of both MZ and DZ twins (Matte *et al.*, 1996; Tagliani-Ribeiro *et al.*, 2011) and that our biological hypothesis is focused on aspects related not only to ovulation but also to fertilization and embryo development, we decided to analyze MZ and DZ twins combined.

Blood genomic DNA was extracted in accordance with Lahiri and Nurnberger (1991). For the *IGF1* (CA)n polymorphism, polymerase chain reactions (PCR) were performed as described by Rosen *et al.* (1998), using the primer sequences 5’ 5`GCTAGCCAGCTGGTGTTATT3’ and 5’ACCACTCTGGGAGAAGGGTA3’. The fragments were analyzed by capillary electrophoresis automatic sequencing in an Applied Biosystems 3500 Genetic Analyzer. In order to test the veracity of the results, one sample from each homozygote pair was sequenced for the following alleles: *IGF1* (CA)19_,_ (CA)20_,_ (CA)21, and (CA)22. The samples were purified with the EXO I and SAP enzymes, and then subjected to Sanger sequencing. In turn, the rs2854744 polymorphism of the *IGFBP3* gene was determined via the TaqMan SNP Genotyping Assay through allelic discrimination using the C_1842665_10 assay in accordance with the manufacturer’s instructions (Applied Biosystems, USA). The reactions were conducted in the StepOnePlus PCR Real-Time System (Applied Biosystems), and the reaction products were analyzed in the StepOne v. 2.2.2 software.

The Hardy-Weinberg equilibrium was calculated using version 3.11 of the Arlequin program. Simple comparisons of *IGF1* allelic frequencies in the cases and controls were done using the *G*-Test (likelihood ratio chi-square), with a 95% confidence interval, in version 11.15 of the WinPEPI program. For analysis of the *IGFBP3* gene, the two-tailed Fisher’s exact test and Pearson’s chi-square tests were used to compare the allelic and genotypic frequencies between case and control groups using IBM SPSS v.18.0 software (IBMCorp., Armonk, NY).

## Results

The case and control groups were in Hardy-Weinberg equilibrium for both of the polymorphisms tested (*p* > 0.05). The analyses of the *IGF1* (CA)n polymorphism revealed eight different alleles in CG, varying from 11 to 22 CA repeats (Table 1). In turn, alleles with 23 and 24 CA repeats (with 200 and 202 bp, respectively) were found in Porto Alegre, but at a low frequency. In all studied populations, the most frequent allele was that with 19 CA repeats, and 192 bp. Although the allele with 22 CA repeats (198 bp) occurred more frequently in the mothers of twins (7.7%) than in both control groups, there was no statistical significance in the distribution of *IGF1* (CA)n between case and control groups from CG (*p* = 0.182) and from PA (*p* = 0.065). Similarly, for the *IGFBP3* rs2854744 polymorphism, there was no statistically significant difference between case and control groups, both for allelic and genotypic frequencies (Table 2).

**Table 1:** Frequency of *IGF1* (CA)n polymorphism in cases and controls.

Number of CA^a^ repeats	Cases		CG^b^ controls				PA^c^ controls	
	(n = 39)		(n = 97)				(n = 117)	
	N	%	N	%	p^d^	N	%	p^d^
11	-	-	1	0.5	0.55	1	0.4	0.55
16	-	-	2	1.0	0.40	2	0.9	0.40
17	1	1.3	3	1.5	0.66	3	1.3	0.97
18	4	5.1	15	7.8	0.37	21	9.0	0.25
19	50	64.1	112	57.8	0.31	148	63.2	0.75
20	13	16.7	47	24.2	0.13	29	12.4	0.38
21	4	5.1	11	5.7	0.86	26	11.1	0.10
22	6	7.7	3	1.5	0.01	2	0.9	< 0.001
23	-	-	-	-	-	1	0.4	0.55
24	-	-	-	-	-	1	0.4	0.55
Total	78	100	194	100	0.18	234	100	0.07

^a^ CA: cytosine-adenosine;

^b^ CG: Cândido Godói;

^c^ PA: Porto Alegre;

^d^ *G*-test

**Table 2:** Frequency of *IGFBP3* rs2854744 gene polymorphism in cases and controls.

Allele	Cases		CG^a^ controls		p^c^	PAb controls		*p* ^*c*^
	(n = 39)		(n = 97)			(n = 117)		
	N	%	N	%		N	%	
C	44	56.4	104	53.6	0.689	112	47.9	0.239
A	34	43.6	90	46.4		122	52.1	
Genotype	N	%	N	%	p^d^	N	%	*p* ^*d*^
CC	14	35.9	32	33.0		32	27.4	
CA	16	41.0	40	41.2	0.927	48	41.0	0.484
AA	9	23.1	25	25.8		37	31.6	

^a^ CG: Cândido Godói;

^b^ PA: Porto Alegre;

^c^ Two-tailed Fisher’s exact test;

^d^ Pearson’s chi-square test

## Discussion

Although IGF-1 has been considered an important factor in the understanding of human twinning (Steinman, 2009), we did not find a statistical association between two genetic polymorphisms commonly related to metabolism in a population with a very peculiar founding history and that shows increased rates of twin births (Matte *et al.*, 1996; Tagliani-Ribeiro *et al.*, 2011).

Interestingly, upon analyzing the results obtained in studies of the *IGF1* (CA)n polymorphism in various continents (Table 3), the frequency of the allele with 22 repeats was found to be low, ranging from 1.5% in European populations (Vaessen *et al.*, 2001; Rietveld, 2009) to 3.4% in a study conducted in China (Xie *et al.*, 2010). In the case group of the present study, this allele occurred at a frequency of 7.7%, while in the controls from both CG and PA, these frequencies were similar to those observed in studies conducted in Europe and North America (Vaessen *et al.*, 2001; Kato *et al.*, 2003; Wen *et al.*, 2005; Cleveland *et al.*, 2006; Rietveld, 2009).

**Table 3:** Percentage of *IGF1* (CA)n polymorphism in general and non-diseased populations.

REGION	BRAZIL	NORTH AMERICA			EUROPE		CHINA	
Number of CA^a^ repeats	This study^b^	Cleveland *et al.* (2006)	Kato *et al.* (2003)	Kato *et al.* (2003)	Rietveld(2009)	Vaessen *et al.* (2001)	Wen *et al.* (2005)	Xie *et al.* (2010)
			White	Black	N = 5386	N = 1080	N = 2172	N = 446
	N = 214	N = 736	N = 112	N = 114				
10	-	-	0.9	-	-	-	-	-
11	0.5	0.1	-	-	-	-	0.1	-
13	-	-	0.9	0.9	-	-	-	-
15	-	-	0.9	9.7	-	0.2	0.2	-
16	0.9	0.3	2.7	3.5	-	0.3	0.1	0.2
17	1.4	1.4	7.1	18.4	1.9	1.9	10.5	7.6
18	8.4	6.0	36.6	25.4	4.6	4.1	16.7	15.9
19	60.7	64.3	30.4	18.4	65.3	65.9	35.1	38.8
20	17.8	18.7	16.1	14.9	19.4	18.7	7.7	6.3
21	8.6	7.5	4.5	6.1	6.9	7.4	26.3	27.1
22	1.2	1.6	-	1.8	1.5	1.5	3.1	3.4
23	0.2	0.1	-	0.9	-	-	0.1	0.5
24	0.2	-	-	-	-	-	-	0.2
Others	-	-	-	-	0.4	-	-	-

^a^ CA: cytosine-adenosine;

^b^ Frequencies of the control samples from Cândido Godói and Porto Alegre

In conclusion, our results indicate a lack of association between twin births in CG and polymorphisms *IGF1* (CA)n and *IGFBP3* rs2854744. Unfortunately, we could not assess serum IGF-1 levels and compare these with the investigated genetic polymorphisms. However, genetic variants related to such factors have been independently associated with several different phenotypes (Wagner *et al.*, 2005; Cleveland *et al.*, 2006; Kaczmarek *et al.*, 2015). Furthermore, to the best of our knowledge, this is the first study to investigate the *IGF1* (CA)n and *IGFBP3* rs2854744 polymorphisms in human twinning. Further investigations in other populations with different characteristics should be performed to better understand the role of IGF-1 in human twin births.
